# Molecular rescue of Tsc1-ablated cortical tuber mice

**DOI:** 10.18632/oncotarget.14239

**Published:** 2016-12-27

**Authors:** Barbara Robens, Albert J. Becker

**Affiliations:** ^1^ Department of Neuropathology, Section for Translational Epilepsy Research, Bonn, Germany

**Keywords:** TSC1, tuberous sclerosis, cortical tuber, in utero electroporation, Neuroscience

Tuberous sclerosis (TSC) is a multi-organic, autosomal-dominant disorder commonly caused by mutations in one of the two tumor suppressor genes *TSC1* or *TSC2* that encode the proteins hamartin and tuberin, respectively. TSC is characterized by highly differentiated malformations in different organs, including cortical tubers in the brain. They occur in the majority of patients and represent the major clinical burden, as they are commonly assumed to be responsible for the severe epilepsy phenotype [1]. While parallel loss of heterozygosity (LOH) is often found in extracerebral TSC associated malformations, such events are only rarely detected in cortical tubers [2]. Independent studies found that classical second hit events in TSC1 heterozygous patients may explain the formation of cortical tubers, while in direct contrast to these findings another group claims that second hits are rare in cortical tubers [1, 3]. Despite the tremendous ongoing research effort on TSC, the exact pathomechanisms leading to the emergence of cortical tubers remain controversial. A major breakthrough for studying the contribution of TSC1 to brain malformations including cortical tubers was the establishment of Tsc1 knockout mice by the Kwiatkowski group [4].

**Figure 1:**
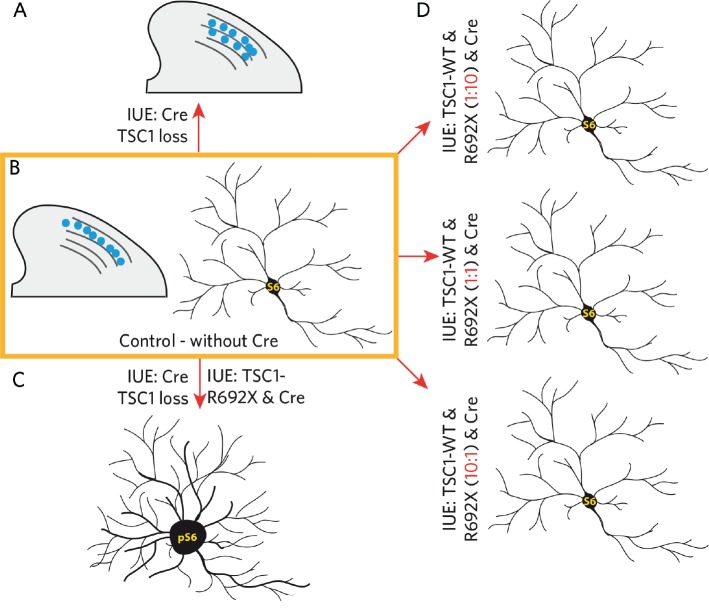
Schematic illustration of Cre-mediated TSC1 loss, consequences of TSC1-R692X co-expression and rescue by varying TSC1-WT plasmid DNA concentrations

In our recently published article [5], we started out from respective transgenic mice to study tuber molecular development aspects [4, 6]. The frequently used C57Bl/6 Tsc1^fl/fl^ mouse line was backcrossed into the CD1 genetic background for at least 3 generations to achieve CD1 Tsc1^fl/fl^ mice. By using this mouse strain we increase pup survival after intraventricular *in utero* electroporation (IUE) dramatically, thus accelerating research on TSC. *In utero* expression of Cre at embryonic day 14 (E14) and subsequent loss of Tsc1 expression in single cells induced cytopathological alterations in mature mice (>P24) reflecting typical tuber-like features: enlarged neuronal cell bodies, increased mTOR activation (assessed by pS6 immunoreactivity), aberrant dendritic arborization and abnormal positioning of cortical neurons (Figure 1A-1C).

To assess whether tuber-like lesions are formed when only one TSC1 allele is lost, we chose an experimental set-up in which Cre is expressed together with the same DNA concentration of a mutated variant of TSC1 (TSC1-R692X - frequently found in TSC1 patients) and the wild type TSC1 (TSC1-WT) to mimic a heterozygous situation. Neurons expressing this plasmid combination showed normal cell size, similar to neurons electroporated with a control plasmid, while expression of only TSC1-R692X together with Cre resulted in a dramatic increase in cell size and thus, substantial mTOR pathway activation. Even very low concentrations of the functional TSC1-WT plasmid relative to the mutated TSC1-R692X plasmid - 1:10 and 1:20 - were sufficient to retain a physiological cellular phenotype indicating a strong rescuing capacity of wild type *TSC1* (Figure 1D). However, we cannot control for the amount of protein that is translated from the plasmid DNAs in our study. We can only assume that the DNA fragments, that are expressed from the same vector backbone with the same promoter, have the same expression efficiency and that the final protein concentration within the Cre-mediated *Tsc1* knockout neurons corresponds to the introduced DNA ratios. On the other hand the experimentally, carefully adjusted TSC1-WT vs. TSC1-R692X protein ratios may be maladjusted by rapid degradation of the mutated TSC1- variant but not the TSC1-WT *in vivo*. But as we see robust and continuous expression of TSC1-R692X-mCherry fusion proteins in other experimental set-ups, we can exclude that the non-functional hamartin^R692X^ is lost in neuronal progenitor cells in our present study.

Despite these experimental limitations, our results suggest robust silencing of the second *TSC1* allele in the emergence of cortical tubers and favor Knudson’s ‘2-hit hypothesis’ as underlying pathogenetic scenario. Technical limitations may explain the TSC cases with no identified mutations since intronic mutations and/or genetic mosaicism are hard to detect [7]. Alternatively, post-translational modifications or other molecular alterations can negatively affect the function of the hamartin/tuberin tumor-suppressor complex that may occur at a dynamic, epigenetic level. Similar interfering mechanisms may be operative in a subset of TSC patients in which no *TSC1* or *TSC2* mutations have been identified.

Our results may provide a promising basis for future gene therapies, as already low amounts of functional *TSC1* appear as sufficient to rescue the cellular consequences of bi-allelic *TSC1* inactivation. Local reconstitution of hamartin in a restricted area of the cortex, or directly within the cortical tuber may represent a possible treatment option here. It is already published that one single injection of AAVs into the cerebral ventricle of newborn mice that lost *Tsc1* expression at E12 in almost all neurons of the brain results in improved clinical features compared to non-injected animals [8]. Survival of injected mice was increased from 22 to 52 days and histologically a normalization of neuron size and mTOR activity was achieved. In this study, Tsc1 is lost in neurons all over the mouse brain and life time was only prolonged, therefore it is possible that treatment with a single injection of hamartin reconstituting AAVs in a more localized pathology - as seen in TSC patients with cortical tubers - may have an even more beneficial effect. Nevertheless, this study shows that gene reconstitution is an effective therapeutic approach in a mouse model of TSC.

Even though systemic and repeated therapy with the mTOR inhibitor everolimus has proven effective in reducing clinical symptoms of TSC patients, targeted gene therapeutic approaches should have the potential of a virtual absence of side effects in contrast to long-term pharmacological treatment. Therefore, new treatment options are required to apply these experimental therapies in the future.
